# Comparison of Hospitalized COVID-19 Patients in Terms of Disease Course According to the Presence of Gastrointestinal Symptoms Before and After Admission

**DOI:** 10.5152/tjg.2023.22003

**Published:** 2023-04-01

**Authors:** Enes Ali Kurt, Oğuz Kağan Bakkaloğlu, Tuğçe Eşkazan, Selma Akdoğan, Uğur Önal, Selçuk Candan, Rıdvan Karaali, Bilgül Mete, Yalım Dikmen, Murat Tuncer, İbrahim Hatemi, Kadir Bal

**Affiliations:** 1 Division of Internal Medicine, Department of Gastroenterology, İstanbul University Cerrahpaşa Faculty of Medicine, İstanbul, Turkey; 2 Department of Internal Medicine, #x0130;stanbul University Cerrahpaşa Faculty of Medicine, İstanbul, Turkey; 3 Department of Infectious Diseases and Microbiology, İstanbul University Cerrahpaşa Faculty of Medicine, İstanbul, Turkey; 4 Department of Anesthesiology and Reanimation, İstanbul University Cerrahpaşa Faculty of Medicine, İstanbul, Turkey

**Keywords:** Diarrhea, anorexia, gastrointestinal symptoms, intensive care, SARS-CoV-2

## Abstract

**Background:**

This study aimed to find the prevalence of gastrointestinal symptoms in hospitalized COVID-19 patients and to investigate the effects of gastrointestinal symptoms on the course of the disease during hospitalization.

**Methods:**

Patients who were hospitalized due to COVID-19 were included in this retrospective study. The diagnostic method of COVID-19 was either a positive reverse transcription polymerase chain reaction test or a typical finding in chest computed tomography. This study was conducted by contacting patients by phone 1 month after they were discharged from hospital to investigate gastrointestinal symptoms. Patients’ laboratory findings at the time of admission, medications they used, and clinical findings were obtained from hospital records retrospectively. Patients with gastrointestinal symptoms were divided into 2 groups according to the start of treatment: pre-treatment and post-treatment groups.

**Results:**

At least 1 gastrointestinal symptom (anorexia, weight loss, diarrhea, nausea, vomiting, and abdominal pain) was present in 67.5% of 435 patients (55.6% male, mean age 52.8). If anorexia and weight loss are excluded, the rate of the presence of at least 1 gastrointestinal symptom is 54%. Gastrointestinal symptoms were present in 48.9% before the initiation of COVID-19 treatment. The most prevalent 3 symptoms were anorexia, weight loss, and diarrhea (56%, 52%, and 35.6%, respectively).

Presence of pre-treatment gastrointestinal symptoms was associated with elevated C-reactive protein levels. Pre-treatment gastrointestinal symptoms were more common in those who received oxygen supply and who were intubated. Resolution of gastrointestinal symptoms takes longer time in those who were admitted to intensive care unit. Weight loss and diarrhea were more common in COVID-19 patients with gastrointestinal symptoms who were intubated than who were not intubated. Abdominal pain was not found to be a significant predictor of disease severity.

**Conclusion: **The prevalence of at least 1 gastrointestinal symptom in hospitalized COVID-19 patients was 67%. The most prevalent symptoms were anorexia, weight loss, and diarrhea. Presence of pre-treatment gastrointestinal symptoms was associated with elevated C-reactive protein levels, use of oxygen supply, and intubation. Gastrointestinal symptoms persist longer in those admitted to intensive care unit.

**Main Points**

- Watery diarrhea was associated with peripheral capillary oxygen saturation <90 and need for intubation.
- Weight loss was associated with intubation and disease severity.
- Abdominal pain was not found to be a significant predictor of disease severity.
- The duration of gastrointestinal symptoms was longer in patients who were treated in intensive care unit.

## Introduction

Gastrointestinal system (GIS) symptoms may develop during COVID-19. There is a high expression of angiotensin-converting enzyme 2 (ACE2), the viral host receptor in the GIS.^1^ Severe acute respiratory syndrome-coronavirus 2 (SARS-CoV-2) may enter the body through the respiratory or GIS. The virus uses the ACE2 receptor which is widely expressed along the intestinal tract to invade the enterocytes rapidly and to multiply.^2^ When SARS-CoV-2 colonizes the GI tract, it may lead to gastritis, enteritis, and colitis.^3^ In addition, since the GI tract is the largest immune organ in the body, it may harbor rapidly multiplying SARS-CoV-2 colonies.^4^ Some investigators claim that the degeneration in GI epithelium layer may lead to the release of endotoxins and microbial metabolites that trigger inflammation and cytokine release in distant organs such as the lungs.^5,6^

In a prospective multinational study, it was reported that the prevalence of GI symptom was 59.7% in hospitalized patients with COVID-19 that was significantly higher than the control group in which the rate was 43.2%.^7^

Still, the COVID-19 pandemic is an evolving process due to newly emerging variants and changes in disease course because of mass population vaccination program. At the beginning of the pandemic, it was reported that GI symptoms can worsen the prognosis of COVID-19,^8-10^ but later it was reported that GI symptoms were associated with a decreased risk of death.^11,12^

A meta-analysis which included articles from December 1, 2019, to May 1, 2021, reported that GI symptoms were not associated with the mortality of COVID-19.^13^ While it was reported in this article that gastrointestinal symptoms adversely affected the prognosis in Asian studies, the opposite was reported by European and American studies.^13^

In a prospective study, it was also reported that COVID-19 which was associated with GI symptoms did not increase the risk of intensive care unit (ICU) admission or mortality. According to this study, the remaining symptoms at 1 month after COVID-19 were nausea and regurgitation.^7^

This study aimed to find the prevalence of GIS symptoms in hospitalized COVID-19 patients and to investigate the association of GI symptoms with the disease course during hospitalization.

## Materials and Methods

This is a retrospective study that included hospitalized patients with COVID-19.

### Case Selection

**Figure 1.**
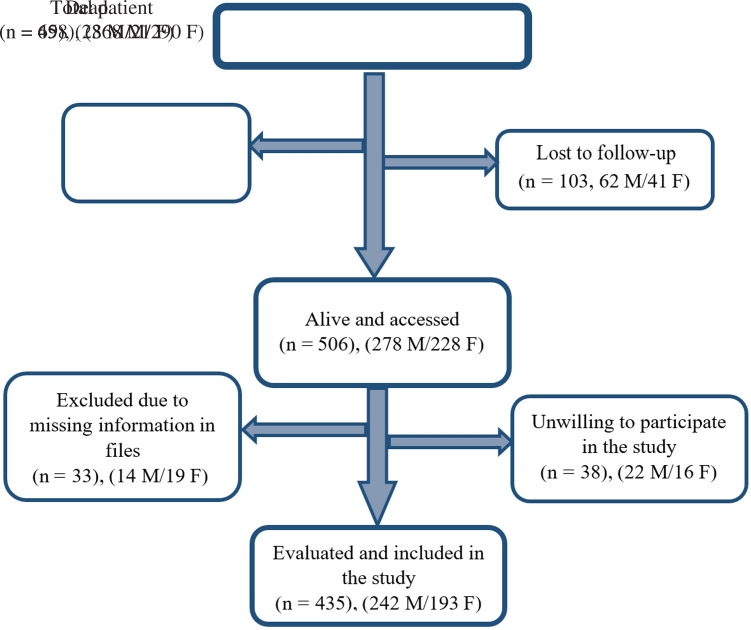
Patient flow chart. M, male; F, female.

A total of 658 hospitalized patients with a diagnosis of COVID-19 diagnosed by polymerase chain reaction (PCR) test and/or chest computed tomography (CT) between March 2020 and June 2020 were identified at İstanbul University Cerrahpaşa Faculty of Medicine, İstanbul, Turkey. Clinical and laboratory data were retrospectively reviewed through the hospital’s electronic data management system. Patients were contacted by phone 1 month after their discharge to inquire about their current status, as well as their symptoms during the disease period. Patient flow chart is shown in Figure 1.

### Patients Who Were Not Included in the Study

We were unable to access 103 (62 males, 41 females) patients (15.6%). We confirmed that they were alive using the death notification system of our country. A total of 49 patients including 28 males had died due to COVID-19. A total of 38 patients (22 males, 16 females) did not want to participate to the study. A total of 33 patients with some missing information in their interviews or their hospital file reviews were excluded. Remaining 435 patients (242 males/193 females) were included in the study.

### Clinical Assessment

A survey form about GI symptoms was created for clinical evaluation and filled out by gastroenterology fellows who interviewed the patients by phone. Anorexia, nausea, vomiting, ageusia, abdominal pain, weight loss, and diarrhea were evaluated as GIS symptoms. Presence of mucous diarrhea, bloody diarrhea, and nocturnal diarrhea was additionally inquired in those who reported diarrhea. Patients were inquired about the presence of COVID-19 in other household members and also about shared use of toilets. Patients were inquired about the presence of any previously known GI diseases. They were asked whether the GIS symptoms occurred before or after the beginning of treatment of COVID-19. The duration of the GIS symptoms accompanying the respiratory symptoms was also noted. The duration of symptoms was classified as shorter than 1 week, 1-2 weeks, and longer than 2 weeks.

### Laboratory and Other Clinical Data

Patients’ biochemical parameters were retrieved from the electronic file system. Treatments received for COVID-19 during hospitalization, as well as hospitalization duration, were recorded. Patients’ blood pressures, heart rates and peripheral oxygen saturation, use of nasal O_2_, use of O_2_ mask with reservoir, use of high-flow oxygen mask, and needs for intensive care and intubation during hospitalization were recorded. For elevated C-reactive protein (CRP) levels, 2 arbitrary cut-offs were established which were 2 and 10 times the normal value. Some clinical and laboratory findings that may be associated with poor prognosis were selected (Supplementary Table 1).

Patients were divided into the following 3 categories according to their admission: 1—those who were brought to the emergency department with an ambulance, 2—those who applied to the outpatient clinic due to their complaints, and 3—those who applied for COVID-19 test due to close contact with a COVID-19 patient although they were not symptomatic.

### Ethics Approval

This study was approved by İstanbul University Cerrahpaşa Faculty of Medicine University Ethics Committee on 2020.07.10 (number: 87841)

Informed consent of the patients was obtained.

### Statistical Analysis

Categorical variables were expressed as number and proportion and were analyzed by chi-square or Fisher’s exact tests. Continuous variables were expressed as mean and standard deviation, and the normality distribution was tested by the Shapiro–Wilk test. Two groups were analyzed by independent samples *t* test for the normally distributed variables and by the Mann–Whitney *U* test for the non-normally distributed variables. We compared more than 2 groups by the one-way analysis of variance for the normally distributed variables and Kruskal–Wallis test for the non-normally distributed variables. Pearson’s or Spearman’s correlation analysis was used to find the relationship between continuous variables, depending on the distribution. Statistical analyses were performed using the International Business Machines Statistical Package for the Social Sciences version 21 Windows software (IBM Corp.; Armonk, NY, USA) and was reported with 95% CIs. Values of *P* < .05 were considered significant.

## Results

Of a total of 435 patients evaluated, 242 were males (55.6%). Mean age of the entire group was 52.8 ± 15.6 years (median, 53; range, 16-90). There was no difference between males and females in terms of mean age. A total of 318 (73%) patients had positive PCR tests, while 418 (96%) patients’ CT showed typical lung involvement. Of the patients with typical lung involvement on tomography, 142 (34%) had a negative result in their first PCR test and 30 (21%) had a positive result in their second PCR test. There was a total of 112 patients (25.7%) who were PCR-negative and diagnosed based on radiological findings. A total of 24 patients (5.5%) were followed up in the ICU. There was no statistically significant difference between PCR-positive and -negative groups in regard to the rate of treatment in ICU (4.2% vs. 8.6%, *P* = .06) and the need for intubation (1.6% vs. 0.8%, *P* = .43).

### Prevalence of Gastrointestinal System Symptoms

At least 1 GIS symptom was present in 67.5% (n = 294) of all patients (n = 435). If weight loss and anorexia are excluded, the rate of at least 1 GIS symptom was 54%.

In 48.9% of the patients, GIS symptoms appeared before receiving any medical treatment. Prevalence of GI symptoms according to patients’ admission type was shown in Table 1. Persons who were tested due to close contact with a COVID-19 patient did not report any pre-treatment GI symptom, and of those patients, only 1 (4.1%) developed post-treatment GI symptom. This rate was significantly low compared to patients who developed post-treatment GI symptoms (37.5%) (*P* < .001).

**Figure 2.**
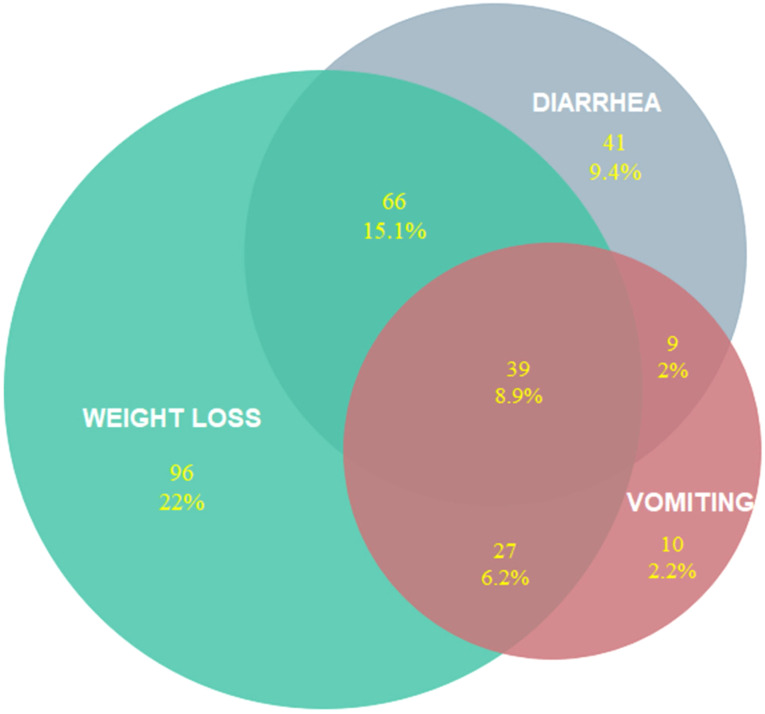
Overlap of 3 main GI symptoms. GI, gastrointestinal.

The most prevalent GIS symptoms were anorexia and weight loss (56% and 52%, respectively) followed by diarrhea (35.6%). The rarest symptom was bloody diarrhea which was reported in 4 patients. Symptoms which were reported at the admission were shown in Table 2. Overlap of the most prevalent symptoms was shown in Figure 2. The rate of diarrhea and ageusia was higher in PCR-positive patients (Table 3).

The number of patients sharing the same toilet with household members was 300 (69%). Coronavirus disease-2019 was also detected in the other household members in 202 (67.3%) of the 300 patients. The number of patients not sharing the same toilet was 135 (31%). Of these patients, household members of 18 (13%) had COVID-19. This difference was statistically significant (*P* < .001).

### Relationship Between the Time of the Onset of Gastrointestinal System Symptoms and Disease Course

There were 14 patients who had GIS symptoms and used oxygen therapy with mask reservoir. All of these 14 patients have had GIS symptoms before the beginning of the treatment (*P* = .013). All 5 intubated patients with GIS symptoms also have had GIS symptoms before the beginning of the treatment. The rate of patients who had symptoms before the beginning of the treatment was significantly higher in patients who had CRP level >50 mg/L (0.014) and who had CRP >10 mg/L (0.039).

### Relationship Between Gastrointestinal System Symptoms and Respiratory System Findings

At least 1 GIS symptom was present in 118 (75.2%) of the 157 patients with fever and 145 (73.6%) of the 197 patients with cough. At least 1 GIS symptom was present in 63.3% of the patients without fever and 62.6% of the patients without cough. The prevalence of GIS symptoms was significantly higher in those with fever and cough (*P* = .011, *P* = .015). On the other hand, no significant relationship was found between dyspnea and GIS symptoms (*P* = .061).

### Gastrointestinal System Symptoms and Drugs Used for COVID-19

The relationship between medications and the onset of GI symptoms was shown in Table 3. The prevalence of any GI symptom was higher in patients receiving tocilizumab and corticosteroid compared to those not receiving these treatments, and 60% and 76% of the patients, respectively, had GI symptoms before these drugs were started. The majority of these patients who received medical treatment already had symptoms before receiving medications (Supplementary Table 2).

78 of the patients receiving hydroxychloroquine and 23 of the patients receiving favipiravir became symptomatic after treatment. The most common side effects were weight loss (66.6% and 73.9%) and diarrhea (61.5% and 65.2%), respectively. The distribution of GI symptoms according to drugs used for COVID-19 is shown in Supplementary Table 3.

The duration of GIS symptoms which lasted longer than 2 weeks was significantly more common in patients who used tocilizumab or corticosteroid (*P *= .026 and *P *= .048, respectively). Details were shown in Table 4.

### Progress of COVID-19 in Patients with Gastrointestinal System Symptom

#### Progress of Gastrointestinal System Symptoms in Intensive Care Patients

Of the 24 (5.5%) patients admitted to intensive care, 17 (70.8%) had a GIS symptom. Comparison of patients with or without intensive care treatment did not reveal any significant difference related to the presence of GIS symptoms. However, all GIS symptoms lasted longer in those who were admitted to ICU. Of the 17 patients with GIS symptoms admitted to intensive care, symptoms regressed in a period shorter than 1 week in 4 patients (23.5%), between 1 and 2 weeks in 2 (11.8%), and in a period longer than 2 weeks in 11 (64.7%). Comparison of symptom durations of 277 (94.2%) GIS symptomatic patients who were not admitted to ICU and 17 (5.8%) GIS symptomatic patients who were admitted to ICU demonstrated that symptoms’ disappearance was longer in those admitted to ICU (*P* < .001).

#### Relationship Between the Duration of Gastrointestinal System Symptoms and Disease Progression

Symptom durations were longer in those who had GI symptoms before treatment (lasting >14 days, *P *= .002). In those who had GI symptoms after treatment, symptom durations were shorter than 1 week and this was significantly more prevalent (symptom disappearance in <1 week, *P* < .001).

Gastrointestinal system symptoms regression was longer in patients with poor prognostic factors including oxygen saturation < 90, need for oxygen mask with reservoir, and admittance to ICU. Symptoms lasted 14 days or longer in 11 (64.7%) of patients with GIS symptoms and who were admitted to ICU (*P* < .001). Symptom duration was longer than 14 days in 50% of the patients using O_2_ mask with reservoir (*P *= .038).

There was a relationship between the duration of GIS symptoms and elevated CRP. Recovery took even longer in those with CRP > 2 × upper limit of normal levels (UNL) (*P *= .026) and CRP >10 × UNL (*P* = .004).

Of a total of 157 GIS symptomatic patients with CRP 10 or more times the normal, symptoms lasted <7 days in 74 (47%), 7-14 days in 41 (26.2%), and 14 days and longer in 42 (26.8%).

Gastrointestinal system symptoms of patients using tocilizumab regressed later compared to those not using it (*P *= .026). In the above-mentioned group, these symptoms returned to normal in 14 days or more in one-third (n = 13) of the 39 GIS symptomatic patients. However, no significant relationship was observed between elevated ferritin (>500) and the duration of GIS symptoms (*P *= .076).

### Presence of Any Known Gastrointestinal System Disease

The number of patients with any known GIS disease was 35 (8%) (Supplementary Table 4).

In the presence of any known GIS disease, although anorexia (*P *= .018), diarrhea (*P *= .012), and weight loss (*P *= .024) were more prevalent, there was no significant difference in the frequencies of nausea, vomiting, ageusia, and abdominal pain. No significant relationship was detected between the time of the onset of the GIS symptoms and the presence of any known GIS disease during the COVID-19 process.

### Gastrointestinal System Symptoms and Biochemical Parameters

The alanine aminotransferase (ALT) and aspartate aminotransferase (AST) values of the 294 patients with GIS symptoms were compared to cases whose values were not >2×UNL. Of the patients, 19 (6.5%) had AST >2×UNL and 14 (4.8%) had ALT >2×UNL. The transaminase values of COVID-19 patients with any GIS symptom were 2×UNL or higher in approximately 5% of these subjects. No significant difference was detected between GIS symptomatic and non-symptomatic patients in terms of elevated transaminases 2×UNL.

### Sub-Groups of Gastrointestinal System Symptoms

Relationships between laboratory and clinical findings and sub-groups of GI symptoms were shown in Tables 5 and 6. C-reactive protein was higher in those with symptoms of anorexia, vomiting, diarrhea, and weight loss compared to those without these symptoms (Table 6). Ferritin values were significantly higher in those with anorexia, diarrhea, and weight loss compared to those without these symptoms (Tables 5 and 6). Peripheral capillary oxygen saturation (SpO_2_) was significantly lower in patients with diarrhea and weight loss higher than 10% compared to those without (Table 6). Of the patients with anorexia, 79.5% also had weight loss. Of those with weight loss, 85% had anorexia. A total of 155 (35.6%) patients had diarrhea, 10% (n = 55) had nocturnal stooling, and 5% (n = 22) had mucous diarrhea, while only 4 patients (1%) had bloody diarrhea.

Based on patients’ pre-COVID weights, their losses with respect to their weights were divided into percentiles. Of the patients, 47% (n = 207) did not have weight loss. Relative to their weight, 29.7% (n = 129) of the patients had a weight loss by <5%, 17.9% (n = 78) had a weight loss by 5%-10%, and 4.8% (n = 21) had a weight loss by 10% and higher. Admittance to intensive care and intubation were more prevalent in patients with weight loss compared to those without weight loss (Table 6). Of a total of 24 patients admitted to ICU, 18 had weight loss; weight loss was by 10% and higher in 7 of these patients. Weight loss was significantly more in those with CRP > 2×UNL (*P *= .035), the loss was even more significant in those with CRP > 10×UNL (*P* < .001). Weight loss was present in 102 (43.4%) patients with a hospitalization duration of <7 days, 124 (62%) patients with a hospitalization duration of 7-14 days, and 37 (71.2%) patients with a hospitalization duration of >14 days. Weight loss increased as hospitalization duration extended (*P* < .001).

## Discussion

The mechanism underlying the GI symptoms of COVID-19 is obscure. Any direct viral cytopathic damage in the mucosa and the indirect effect of COVID-19’s systemic cytokine storm may both contribute to GI symptoms.^14^ Despite the increasing number of publications regarding COVID-19, the significance of GI symptoms is still unclear. The reason for this is multifactorial. First of all, the majority of studies on COVID-19 are retrospective and depend on electronic medical records collected in an urgent setting far from optimal conditions. In addition to the inevitable limitations of electronic medical records, the lower priority of GI symptoms against severe respiratory distress syndrome may lead to under-recording or even not-recording of GI symptoms. Secondly, diarrhea and nausea, which are among the most common digestive symptoms of COVID-19, are generally evaluated subjectively and their definitions are open to misinterpretations particularly in emergency situations. Both nausea and diarrhea may occur due to multiple drugs such as certain antibiotics or antivirals, as well as enteral feeding. Also, preexisting GI conditions may worsen GI symptoms. Considering all of the above, this study was based on the inquiries about GI symptoms performed by conducting phone interviews with patients after their discharge due to hospitalization for COVID. The authors want to notice that receiving information over the phone interview is subject to handicaps due to the patient’s misremembering or forgetting certain things about their symptoms.

Within the entire group, prevalence of any GI symptom during diagnosis was 67%. Gastrointestinal symptoms developed in 18% of the patients after treatment. Patients who had no common symptoms related to COVID-19 such as fever, myalgia, or cough and who were diagnosed during screening did not have any GI symptoms. The prevalence of post-treatment GI symptoms (4%) in these patients was lower compared to that of symptomatic patients (19.4%). The most prevalent GIS symptoms were anorexia (56%) and weight loss (52%). In approximately 80% of the patients, symptoms of anorexia and weight loss were concurrent. Another prevalent symptom was diarrhea (35.6%). In 15% of the patients, diarrhea and weight loss were concurrent. According to a multicenter cohort including 318 patients^15^, 61.3% of the patients reported at least 1 gastrointestinal symptom at admission, the most common being anorexia (34.8%), diarrhea (33.7%), nausea (26.4%), and weight loss (9.4%). In our study, the concurrency of the 3 main GI symptoms including diarrhea, weight loss, and vomiting was 9%. Accompanying anorexia and weight loss in patients with GI symptoms such as diarrhea and vomiting may be explained by the disease affecting the GI system.

Although symptoms emerged before hospitalization in approximately 70% of the patients, the hospital environment may still be associated with anorexia and weight loss. Limited food options and pre-set meal timing in the hospital setting, as well as certain drugs used during hospitalization, may also cause GI symptoms. In our study, no relationship was detected between medication and GI symptoms. Considering the time of onset of the symptoms, we can argue that GI symptoms are rather associated with infection instead of the drug treatment because symptoms that started before treatment, in other words which can be associated with COVID-19, lasted longer compared to symptoms that started after medical treatment. It is difficult to establish any relationship between GI symptoms and COVID since it is known that the main factors determining the progress of COVID-19 are associated with respiratory system findings and systemic inflammatory response. Hospitalization duration was longer in patients with diarrhea, nausea, vomiting, anorexia, and weight loss. While this may be related to the virus load, it may also be associated with its effects on the other systems.

In this study, watery diarrhea was associated with SpO_2_ <90 and need for intubation. Dehydration, malabsorption, decreased absorption of oral drugs, and weight loss may affect the progression of COVID-19. Corticosteroid and tocilizumab use was also more prevalent in those with diarrhea, which may be an indirect sign of increased inflammatory response in those with diarrhea. Weight loss was the second symptom which was associated with intubation. The etiology of weight loss in COVID-19 is multifactorial and presumably develops due to increased catabolism and anorexia related to the severe inflammatory response, as well as decreased intake and diarrhea. In a previous study,^16^ 31% of hospitalized patients had lost >5% of their initial body weight. C-reactive protein levels at admission were also significantly higher in the weight loss group. The fact that weight loss is the only GI symptom associated with admittance to intensive care may be due to its association with inflammatory response. Gastrointestinal symptoms of patients requiring intensive care lasted longer. Presumably, patients undergoing a more severe COVID-19 have more severe and sustained GI symptoms.

In contrast to other studies,^15,17^ abdominal pain was not found to be a significant predictor of disease severity in our study. C-reactive protein levels were higher in those with anorexia, vomiting, diarrhea, and weight loss compared to those without these symptoms. Weight loss and diarrhea were the GI symptoms associated with the cut-off value determined as CRP >50 mg/L.

It is known that COVID may be found in the stool and claimed that toilet hygiene is important in terms of transmission. Our study also demonstrated that the rate of COVID-19 was higher in household members using the same toilet as the patient. However, the data should be interpreted carefully as the risk of airborne infection is high in poorly ventilated indoor areas. A definitive conclusion cannot be reached on this matter since we do not have any data on the presence of the virus in the stool.

We believe that by conducting phone surveys with the patients, we were able to gather more information regarding GI symptoms than those previously recorded; however, as is the case in any similar study, there remains the possibility of subjective evaluation by the patients and they may have recalled incorrectly or forgotten some of the information. Furthermore, inaccessible patients and patients who were accessed but who refused to participate in the study also contributed to data loss. Another issue is the exclusion of the deceased patients. Although certain information can be obtained regarding GI symptoms from the file data of the deceased patients, as explained above, we thought that this would not be sufficient. Therefore, this study does not include any data regarding the relationship of GI symptoms with mortality which is actually the most significant endpoint in terms of prognosis. Another issue is the inclusion of only hospitalized patients which results in choosing more severe patients. Because of that, our results are valid only for hospitalized patients. Another weak point is the definition of COVID-19 in our group. During pandemic, some patients were treated according to their radiological and clinical findings without demonstration of PCR positivity. This is a selection bias, but we can argue that our results are real-life data. Because of exclude PCR negative patients would also create a selection bias. In addition, there was no difference between PCR-positive and -negative in regard to intensive care unit treatment and in intubation rate. On the other hand, the rate of diarrhea and ageusia was more common in PCR-positive patients.

In summary, the most prevalent symptoms in hospitalized patients due to COVID-19 were weight loss and anorexia. These symptoms, which were most of the time concurrent, were followed by watery diarrhea. Weight loss and diarrhea were more prevalent in intubated patients. These symptoms may be indirect indicators of disease severity in hospitalized COVID-19 patients.

## Figures and Tables

**Table 1.** Distribution of the Presence of GI Symptoms According to the Method of Admission of the Patients

Admission Type	Presence of GI Symptoms Before Treatment, n (%)	Presence of GI Symptoms After Treatment, n (%)	Presence of GI Symptoms, n (%)
Asymptomatic patients screened due to close contact (n = 24)	0 (0)	1 (4.1)	1 (4.1)
Admission to the outpatient clinic with symptoms (n = 312)	161 (51.6)	70 (22.4)	231 (74)
Admission to the emergency department with ambulance (n = 99)	52 (52.5)	10 (10.1)	62 (62.6)
Total (n = 435)	213 (48.9)	81 (18.6)	294 (67.5)

GI, gastrointestinal.

**Table 2.** Distribution of Symptoms at Admission

Weakness	307 (70.5%)	Anorexia	244 (56.1%)
Fever	291 (66.8%)	Weight Loss	228 (52.4%)
Myalgia	283 (65%)	Diarrhea	155 (35.6%)
Cough	244 (56%)	Watery	129 (29.6%)
Dyspnea	193 (44.3%)	Mucous	22 (5.1%)
Sputum	68 (15.6%)	Bloody	4 (0.9%)
Headache	57 (13.1%)	Ageusia	150 (34.5%)
Nasal congestion	45 (10.3%)	Nausea	147 (33.8%)
		Vomiting	85 (19.5%)
		Abdominal pain	63 (14.4%)
		Epigastric	32 (7.3%)
		Diffuse	31 (7.1%)

**Table 3.** The Prevalence of Gastrointestinal Symptoms According to PCR Test Positivity

	PCR (+)	PCR (−)	*P*
Anorexia	57%	53.9%	.31
Weight loss	52.1%	53.1%	.9
Diarrhea	38.8%	28.1%	**.022**
Ageusia	37.8%	26.6%	**.016**
Nausea	34.5%	32%	.35
Vomiting	19.9%	18.8%	.45
Abdominal pain	14.6%	14.8%	.9

PCR, polymerase chain reaction.

*P*<0.05 were bold values.

**Table 4.** The Time of Onset of the GI Symptom and the Duration of the GI Symptom

	<1 Week, n (%)	1-2 Week, n (%)	>2 Week, n (%)	*P*
GI symptoms before diagnosis (n = 213)	104 (48.8)	53 (24.9)	56 (26.3)	Between groups of GI symptoms before and after diagnosis >2-week vs. < 2-week *P* = .002 <1-week vs. > 1-week *P* < .001
GI symptoms after diagnosis (n = 81)	60 (74.1)	13 (16)	8 (9.9)	

GI, gastrointestinal.

**Table 5.** GI Symptoms and Biochemical Values

	Anorexia			Nausea			Vomiting			Abdominal Pain			
	Present	Absent	*P*	Present	Absent	*P*	Present	Absent	*P*	EPI	DIFF	Absent	*P*
	Present	Absent	*P*	Present	Absent	*P*	>10%	5%-10%	<5%	Absent	*P*		
**CRP mg/L **	66.3 (105)	33.2 (78)	**.001**	59 (116)	48.6 (83)	.268	61 (135)	48.6 (83)	**.046**	51.1 (70.6)	48.3 (137)	53 (89)	.916
**LYMP /mm ^3^**	1.4 (0.9)	1.5 (0.9)	.161	1.4 (1)	1.4 (0.9)	.671	1.3 (1.2)	1.5 (0.8)	.524	1.2 (0.6)	1.4 (1.1)	1.5 (0.9)	**.027**
**d -Dimer µg/mL**	1.22 (3)	1.21 (2)	.298	1.15 (2)	1.33 (2)	.224	1.31 (2)	1.16 (2)	.399	0.98 (2)	1.2 (2)	1.24 (2)	.520
**Ferritin nq/mL**	366 (624)	275 (439)	**.006**	324 (527)	302 (524)	.739	353 (557)	294 (509)	.290	341 (484)	310 (566)	307 (521)	.971
**LDH U/L**	342 (222)	306 (271)	.055	327.5 (229)	327 (267)	.988	341 (227)	316 (256)	.115	349 (228)	317 (273)	327 (256)	.936
**CR mg/dL**	0.97 (0.35)	1.02 (0.41)	.160	0.95 (0.32)	1.01 (0.4)	.161	0.97 (0.34)	0.99 (0.4)	.863	0.96 (0.56)	0.96 (0.31)	0.99 (0.38)	.705
**ALT U/L**	22.4 (17.9)	19.3 (17.7)	.116	20.4 (16.2)	20.8 (18.2)	.889	18.5 (15.3)	20.9 (18)	.467	17 (18.8)	18 (10.7)	21 (19)	.412
**AST U/L**	25 (13.8)	22.2 (12.9)	**.028**	22.4 (16.9)	23.3 (16.7)	.408	23.2 (17.1)	23 (17)	.794	21.6 (21.5)	25 (13.3)	23.2 (17.2)	.790
**SO _2_**	92 (7)	94 (7)	.192	93 (7)	93.5 (7)	.421	92 (6)	93.5 (7)	.130	92 (7)	93 (6)	93 (7)	.965
	Diarrhea			Mucous D (Subgroup)			Weight Loss						
**CRP mg/L **	61.3 (118)	46.1 (83)	**.039**	43.4 (131)	53 (90.3)	.658	140.9 (190)	70.7 (70.1)	64.7 (94.8)	33.6 (83)	**.000**		
**LYMP /mm ^3^**	1.4 (0.8)	1.5 (0.9)	.149	1.2 (0.8)	1.4 (1)	**.049**	1.4 (1.1)	1.3 (0.9)	1.4 (0.7)	1.5 (0.9)	.240		
**d -Dimer µg/mL**	1.43 (3)	1.14 (2)	.179	1.42 (3)	1.21 (2)	.772	3.3 (15)	1.45 (3)	1.09 (3)	1.17 (2)	**.012**		
**Ferritin nq/mL**	409 (515)	282 (463)	**.015**	437 (755)	300 (522)	**.047**	727 (868)	398 (534)	369 (496)	245 (446)	**.000**		
**LDH U/L**	365 (261)	309 (244)	**.007**	368 (335)	327 (248)	.333	622 (447)	390 (236)	328 (206)	287 (239)	**.000**		
**CR mg/dL**	0.99 (0.41)	0.98 (0.38)	.813	0.96 (0.44)	0.99 (0.38)	.959	1.03 (0.51)	1 (0.36)	0.96 (0.36)	0.98 (0.41)	.940		
**ALT U/L**	20.2 (16.2)	20.7 (18.4)	.821	22.5 (18.6)	20.4 (17.8)	.723	17.7 (17)	21.8 (18.1)	24.1 (16.8)	19.3 (18.6)	.626		
**AST U/L**	22.4 (16.3)	23.1 (16.9)	.818	26 (24)	23 (16.5)	.424	26.6 (23.8)	25.2 (28.7)	24.3 (18.3)	22.6 (12)	.163		
**SO _2_**	91.5 (7)	94 (6)	**.004**	90.5 (8)	93 (7)	.498	89 (7)	92 (7)	93 (7)	94 (7)	**.009**		

Numbers in the columns of present and absent are median and (IQR).

IQR, interquartile range; LYMP, lymphocyte; CR, creatinine; ALT, alanine aminotransferase; AST, aspartate aminotransferase; SO_2_, oxygen saturation; EPI, epigastric pain; DIFF, Diffuse abdominal pain; mucous D, mucous diarrhea; GI, gastrointestinal.

*P*<0.05 were bold values.

**Table 6.** GI Symptoms and Poor Prognostic Parameters, Hospitalization Duration

n,*P*	Anorexia (n = 244)	Nausea (n = 146)	Vomitting (n = 85)	Abdominal Pain (n = 63)	Diarrhea		Ageusia (TasteLoss) (n = 149)	Weight Loss (>%5 Total) (n = 97)
					Watery (n = 129)	Mucous (n = 22)		
SpO2 < 90	27% (0.757)	28.7% (0.526)	30.5% (0.429)	20.6% (0.19)	41.8% **(0.003)**	27.2% (0.826)	26.1% (0.945)	31.9% (0.18)
Heart rate >120/min	3.2% (0.287)	4.1% (0.169)	5.8% **(0.049)**	4.7% (0.234)	2.3% (0.37)	0 (0.536)	1.3% (0.185)	5.1% **(0.017)**
Blood pressure < 90/60 mmHg	1.2% (0.426)	2% (0.121)	2.3% (0.183)	0 (0.504)	2.3% (0.131)	0 (0.812)	0 (0.188)	1% (0.657)
Steroid use	5.7% (0.317)	6.1% (0.368)	9.4% **(0.028)**	6.3% (0.374)	10.8% **(0.002)**	4.5% (0.712)	3.3% (0.292)	8.2% (0.086)
Tocilizumab use	11.8% (0.906)	16.4% **(0.033)**	12.9% (0.697)	12.6% (0.835)	19.3% **(0.034)**	22.7% (0.101)	12% (0.897)	18.5% **(0.023)**
Mask with reservoir-mask with high flow	4.9% **(0.023)**	5.4% (0.06)	5.8% (0.121)	6.3% (0.156)	6.2% (0.088)	4.5% (0.522)	40% (0.503)	**7.2% (0.013)**
Need for ICU	6.5% (0.283)	4.1% (0.349)	8.2% (0.221)	3.1% (0.285)	7.7% (0.525)	9% (0.346)	40% (0.315)	11.3% **(0.006)**
Need for intubation	1.6% (0.599)	1.3% (0.672)	2.3% (0.333)	1.5% (0.617)	3.8% **(0.014)**	4.5% (0.269)	1.3% (0.658)	4.1% **(0.026)**
Hospitalization duration (day)	8.6 ± 5.3 SD **(0.016)**	8.9 ± 5.3 SD **(0.010)**	9.4 ± 5.9 SD **(0.006)**	8.5 ± 5.5 SD (0.89)	9.6 ± 6.2 SD **(<0.001)**	8.6 ± 5.9SD (0.9629)	8.0 ± 4.5SD (0.778)	10.1 ± 6.2 SD **(<0.001)**
CRP > 50 mg/L (10×UNL)	59.4% **(<0.000)**	55.4% (0.267)	60% (0.076)	50.7% (0.811)	67.4% (0.114)	45.4% (0.568)	53% (0.622)	68% **(<0.000)**
Ferritin > 500 nq/mL	35.4% **(0.037)**	32.1% (0.57)	35.2% (0.68)	30.1% (0.519)	48% **(0.017)**	40.9% (0.392)	31.5% (0.784)	49.4% **(<0.000)**
Lymphocyte < 500/mm^3^	3.2% (0.617)	3.4% (0.827)	2.3% (0.365)	4.7% (0.427)	2.3% (0.118)	0 (0.429)	0.6% **(0.01)**	3% (0.487)
Platelets < 100 10^3^/mm^3^	2% (0.695)	2% (0.547)	3.5% (0.308)	3.1% (0.446)	2.3% (0.496)	0 (0.592)	0.6% (0.089)	3% (0.407)
d-dimer ≥ 4×UNL	35.2% (0.422)	29.4% (0.104)	30.5% (0.421)	28.5% (0.322)	45.7% (0.174)	27.2% (0.52)	30.8% (0.327)	42.2% **(0.039)**
Creatinine > 1.4 mg/dL	13.1% (0.11)	13% (0.272)	15.2% (0.949)	15.8% (0.98)	19.3% (0.822)	18.1% (0.464)	10.7% **(0.044)**	12.3% (0.285)

Numbers in the columns are % and p.

SpO2, oxygen saturation; CRP, C-reactive protein; ICU, intensive care unit; GI, gastrointestinal.

*P*<0.05 were bold values.

**Supplementary Table 1.** Factors That Can Predict Poor Prognosis

	Poor Prognostic Factors
Baseline clinical parameters	SPo_2_ (in room air) <90 Heart rate >120/min Blood pressure <90/60 mm Hg
Baseline biochemical parameters	CRP >50 mg/L (10×UNL), ferritin >500 ng/mL, lymphocyte <500/mm^3^ Creatinine >1.4 mg/dL, platelets <100 10^3^/mm^3^, d-dimer 4×UNL
Parameters related to treatment modality	Tocilizumab—steroid use
Primary end-points	Need for Mask with reservoir—mask with high flow Need for ICU Need for intubation

CRP, C-reactive protein; ICU, intensive care unit; UNL, upper limit of normal levels.

**Supplementary Table 2.** Drugs and GI Symptoms

Medical Treatment	Presence of GI Symptoms					GI Symptoms Recover Time
	Total %	*P*	Before Treatment %	After Treatment %	*P *	
Hydroxychloroquine (n = 411)	66.9	.411	47.9	18.9	.297	<1 week: 55% 1-2 weeks: 22.5% >2 weeks: 22.5%
Lopinavir/ritonavir (n = 27)	88	.533	51.8	37.1	.106	<1 week: 66.6% 1-2 weeks: 16.6% >2 weeks: 16.6%
Favipiravir (n = 161)	73.2	.055	59	14.2	**.011**	<1 week: 47.5% 1-2 weeks: 25.4% >2 weeks: 27.1%
Tocilizumab (n = 51)	76.4	**.026**	60.8	15.6	.291	<1w: 35.9% 1-2 weeks: 30.8% >2 weeks: 33.3%
Corticosteroid (n = 21)	85.7	**.048**	76.2	9.5	.171	<1 week: 44.4% 1-2 weeks: 11.1% >2 weeks: 44.4%

GI, gastrointestinal.

**Supplementary Table 3.** The Distribution of GIS Symptoms According to Drugs Used for COVID-19

Side Effects	Hydroxychloroquine (n = 78)	Favipiravir (n = 23)	Lopinavir/Ritonavir (n = 10)	Tocilizumab (n = 8)
n (%)				
Anorexia	45 (57.6)	14 (60.8)	6 (60)	3 (37.5)
Diarrhea	48 (61.5)	15 (65.2)	8 (80)	4 (50)
Mucous	1 (1.2)	1 (0.4)	1 (10)	1 (12.5)
Nocturnal stooling	7 (8.9)	2 (0.8)		
Vomiting	14 (17.9)	5 (21.7)	1(10)	1 (12.5)
Nausea	35 (44.8)	9 (39.1)	4 (40)	4 (50)
Abdominal pain	4 (5.1)	1 (4.3)		
Ageusia	30 (38.4)	7 (30.4)	1 (10)	3 (37.5)
Weight loss	52 (66.6)	17 (73.9)	6 (60)	5 (62.5)

GIS, gastrointestinal system.

**Supplementary Table 4.** Known GI Diseases

GI Disease (n = 35)	n (%)
Dyspepsia/gastritis	19 (54)
GERH	7 (20)
Peptic ulcer	3 (8.5)
Cholelithiasis	2 (5.7)
IBD	3 (8.5)
Crohn’s disease	2 (5.7)
Ulcerative colitis	1 (2.8)
Cirrhosis	1 (2.8)

GERD, gastroesophageal reflux disease; IBD, ­inflammatory bowel disease; GI, gastrointestinal.
